# Internal Medicine residents use heuristics to estimate disease probability

**Published:** 2015-12-11

**Authors:** Sen Han Phang, Pietro Ravani, Jeffrey Schaefer, Bruce Wright, Kevin McLaughlin

**Affiliations:** 1 Department of Medicine, University of Calgary, Calgary, Alberta; 2 Office of Undergraduate Medical Education, University of Calgary, Calgary, Alberta

## Abstract

**Background:**

Training in Bayesian reasoning may have limited impact on accuracy of probability estimates. In this study, our goal was to explore whether residents previously exposed to Bayesian reasoning use heuristics rather than Bayesian reasoning to estimate disease probabilities. We predicted that if residents use heuristics then post-test probability estimates would be increased by non-discriminating clinical features or a high anchor for a target condition.

**Method:**

We randomized 55 Internal Medicine residents to different versions of four clinical vignettes and asked them to estimate probabilities of target conditions. We manipulated the clinical data for each vignette to be consistent with either 1) using a representative heuristic, by adding non-discriminating prototypical clinical features of the target condition, or 2) using anchoring with adjustment heuristic, by providing a high or low anchor for the target condition.

**Results:**

When presented with additional non-discriminating data the odds of diagnosing the target condition were increased (odds ratio (OR) 2.83, 95% confidence interval [1.30, 6.15], *p* = 0.009). Similarly, the odds of diagnosing the target condition were increased when a high anchor preceded the vignette (OR 2.04, [1.09, 3.81], *p* = 0.025).

**Conclusions:**

Our findings suggest that despite previous exposure to the use of Bayesian reasoning, residents use heuristics, such as the representative heuristic and anchoring with adjustment, to estimate probabilities. Potential reasons for *attribute substitution* include the relative cognitive ease of heuristics vs. Bayesian reasoning or perhaps residents in their clinical practice use *gist* traces rather than precise probability estimates when diagnosing.

## Introduction

In clinical practice, when we encounter a new case we immediately begin to apply knowledge stored in implicit and/or episodic memory, typically via some type of *heuristic*.^1^–^3^ There are many different types of heuristics, and opinions on what constitutes a heuristic, but according to Gigerenzer and Gaissmaier, a heuristic is a cognitive strategy “…that ignores part of the information, with the goal of making decisions more quickly, frugally, and/or accurately than more complex methods.”^4^ Application of heuristics in the first part of the diagnostic process is typically automatic (*System 1* processing) and leads to the generation of initial diagnostic hypotheses.^1^,^5^,^6^ As additional clinical data are gathered, we may revise our initial hypothesis via the same or different heuristics, or by analytic information processing (*System 2* processing).^7^–^11^ System 2 processing involves semantic memory, the part of long-term memory where we store structured and symbolic representation of knowledge, typically in the form of rules.^2^,^12^ In contrast to System 1, System 2 processing is conscious and effortful, and common examples of this include applying logic or probabilistic reasoning when diagnosing.

System 1 and System 2 processing have both advantages and limitations. For example, using heuristics allows us to generate hypotheses with incomplete data, and makes diagnosing less effortful and more efficient.^4^,^13^ But the literature on *heuristics and biases* suggests that the use of heuristics is error prone, and heuristic use is frequently implicated in cases of diagnostic error.^14^–^16^ To counter the risk of diagnostic error, we typically encourage learners to consciously analyze their diagnostic hypotheses, for example by Bayesian reasoning.^17^–^19^ By applying Bayes’ theorem we can estimate the probability of a given disease from the product of prevalence and the likelihood ratio of disease given the combination of clinical and laboratory findings.^20^ In theory, Bayesian reasoning should result in a more accurate estimation of disease probability, but in practice training in Bayesian reasoning often has limited impact on the accuracy of probability estimates.^21^–^23^ Consistent with this, in a recent study we found that post-test probability estimates of Internal Medicine residents with prior training in Bayesian reasoning were significantly different from literature-derived probabilities.^24^ Several possible explanations were considered for the inaccurate probability estimates in our previous study – including errors in estimating prevalence and likelihood ratios, faulty calculations, or attribute substitution (where a complex cognitive task is replaced with an easier one, such as a heuristic^6^) – but our observational design did not allow us to differentiate between these explanations.

Given the pervasive application of heuristics in human decision-making,^25^ it is possible that despite prior training on how to apply Bayesian reasoning, residents may still use heuristics to estimate disease probabilities.^6^ As heuristics are often applied subconsciously, their use is generally inferred from the results of experimental manipulations rather than from asking participants to think aloud. For example, if a representative heuristic is used to predict probability then adding prototypical clinical features to the case should increase the estimated probability – even if these features are non-discriminating.^25^ An example of a non-discriminating prototypical clinical feature is the presence of obesity in patients with Cushing’s syndrome. Obesity is an expected finding in Cushing’s, but because obesity is very common and Cushing’s is rare, the vast majority of obese individuals do not have Cushing’s. The positive likelihood ratio of 0.1 means that Bayesian estimate of Cushing’s is not increased by the presence of obesity.^26^ Similarly, to uncover another commonly used heuristic – anchoring with adjustment – we could use high or low anchors to suggest that the prevalence of the target condition (or a competing diagnosis) is very high or very low. Prevalence anchors are typically implausible and should have no impact of Bayesian estimates, but may affect post-test probabilities if participants are unable to adjust fully from the suggested prevalence.^25^

Building on the findings of our previous study, our objective in the present study was to explore whether Internal Medicine residents previously exposed to training in Bayesian reasoning use heuristics to generate probability estimates. To do so we manipulated clinical vignette data in two ways: first, to detect the use of a representative heuristic we randomly allocated non-discriminating prototypical clinical features to vignettes; and, second, to identify anchoring with adjustments we assigned high or low anchors to vignettes. We predicted that if residents use heuristics then post-test probability estimates of a target condition would increase with the addition of non-discriminating clinical features or a high anchor for this condition.

## Methods

### Participants

Our participants were 55 residents in the Internal Medicine residency training program at the University of Calgary. All of the residents were in their core training (years 1 to 3) in the program, and most were Canadian medical school graduates who had received training in Bayesian reasoning in their undergraduate program. The extent of formal and informal training in Bayesian reasoning was, however, highly variable and difficult to quantify. The Conjoint Health Research Ethics Board at the University of Calgary approved our study and we obtained informed written consent from all participants prior to randomization.

### Materials

**Figure 1:**
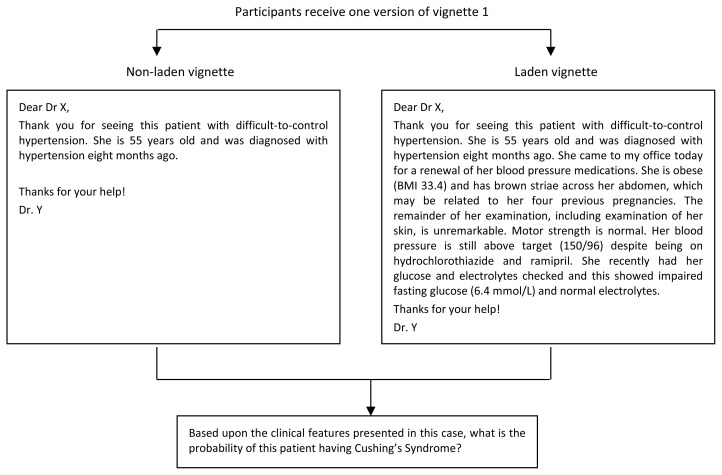
Non-laden and laden versions of vignette 1

We created two study booklets, each containing four clinical vignettes in the form of a referral letter from a primary care physician. For our vignettes we selected four target conditions that are familiar to general internists and for which there are published data on prevalence and likelihood ratios for associated clinical findings (Cushing’s syndrome, hyperthyroidism, peripheral vascular disease, and chronic obstructive pulmonary disease).^22^–^26^ We manipulated the clinical data for each vignette to be consistent either with 1) using a representative heuristic, by adding non-discriminating prototypical clinical features of the target condition (vignettes 1 and 3), or 2) using an anchoring with adjustment heuristic, by providing a high or low anchor for the target condition (vignettes 2 and 4). For vignettes 1 and 3, we created two versions: one containing only demographic information and referring clinical presentation (*non-laden* vignette), and one containing identical demographic information and referring clinical presentation but *laden* by the addition of non-discriminating prototypical clinical features. The non-laden and laden versions of vignette 1 are shown in Figure 1.

For vignette 2, where the target condition was hyperthyroidism, we preceded the description of the case with either a high or low anchor. For the high anchor participants were asked to rate their level of agreement on a five point Likert scale (with descriptors ranging from *very low* to *very high*) with the following statement: “In females under the age of 30 with weight loss of > 10% the prevalence of hyperthyroidism is >95%”, whereas those exposed to the low anchor were asked to rate their agreement with the statement: “In females under the age of 30 with weight loss of > 10% the prevalence of hyperthyroidism is <0.000001%”. For vignette 4, where the target condition was chronic obstructive pulmonary disease, our anchors related to the probability of a competing diagnosis (cardiac failure). Thus the high anchor was: “In individuals over the age of 65 who smoke, the prevalence of cardiac failure is <0.000001%”, and the low anchor was: “In individuals over the age of 65 who smoke, the prevalence of cardiac failure is >95%”.

At the end of each vignette we provided a rating scale for participants to rate the probability of the target condition being the underlying diagnosis in this patient. The rating scale had ten categories: <1%, 1–5, 6–15, 16–30, 31–50, 51–69, 70–84, 85–94, 95–99, and > 99%.

### Procedure

**Figure 2:**
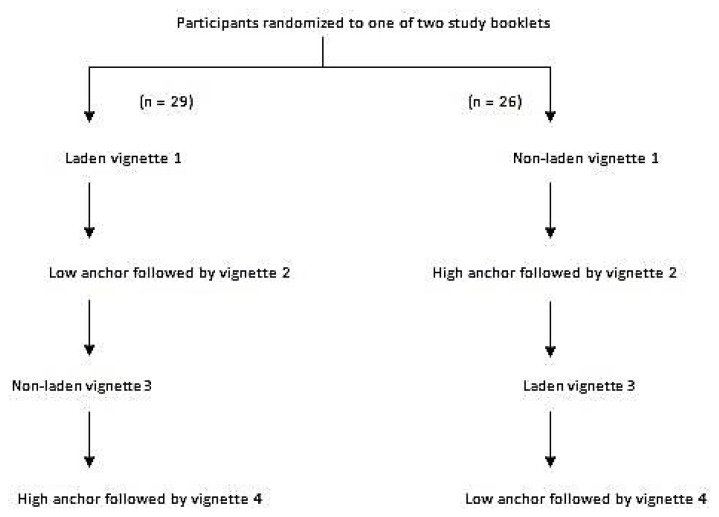
Study design

Using a computer generated random sequence for numbers 1 to 12 (corresponding to the months of the year), we assigned our participants to receive one version of the study booklet according to their birth month. Figure 2 shows our study design along with the version of the vignette in each booklet. During the study our participants did not have access to a computer or other educational resources and did not discuss the vignettes with each other. All participants completed the vignettes in the same order and we allowed 90 seconds to complete the rating of probability of the target condition for each vignette. We used the midpoint of the category selected as the post-test probability of the target condition for each vignette.

### Statistical analyses

For each vignette we converted the crude probabilities into log odds and then used linear regression to compare log odds as a function of the intervention (laden vs. non-laden vignette or high vs. low anchor). We then exponentiated the differences in log odds and their 95% confidence intervals to generate the odds ratio associated with the different types of intervention. We used Stata version 11.0 (StataCorp, College Station, Texas) and R version 3.0.2 (The R Foundation for Statistical Computing) for our statistical analyses.

## Results

**Figure 3:**
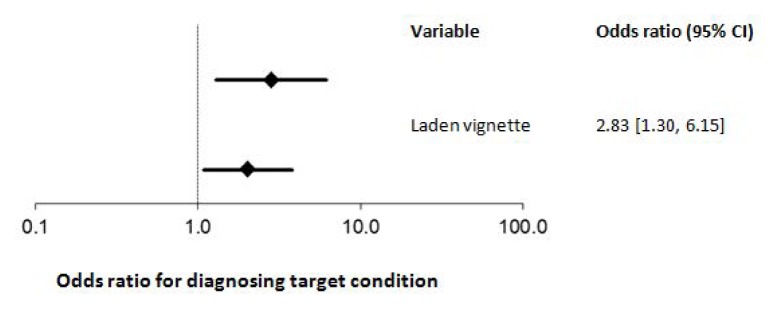
The impact on enhancing clinical vignettes with non-discriminating prototypical features or using anchors on the odds of Internal Medicine residents diagnosing a target condition

When we presented our participants with a vignette laden with non-discriminating information the odds of them diagnosing the target condition were significantly higher than when presented with a non-laden vignettes (odds ratio (OR) 2.83, 95% confidence interval [1.30, 6.15], *p* = 0.009). Similarly, the odds of diagnosing the target condition were also increased when we preceded a vignette with a high anchor vs. low anchor (OR 2.04, [1.09, 3.81], *p* = 0.025). These data are shown in Figure 3.

## Discussion

To offset the perceived limitations of heuristics,^1^,^15^,^16^ many medical schools train students and resident on analytical clinical reasoning strategies, such as the application of Bayes’ theorem to generate accurate post-test probabilities. Yet, prior studies have shown that when students and physicians are enabled to perform Bayesian reasoning (e.g., by presenting data on prevalence and likelihood ratios along with computer resources to perform the appropriate calculations) they often fail to use these correctly to generate accurate probability estimates.^21^–^23^ There are two possible explanations for the failure of Bayesian reasoning to improve accuracy of probability estimates: faulty application of Bayes’ theorem (e.g., due to inaccurate data or calculation errors), or attribute substitution, where alternative cognitive strategies, such as heuristics, are used to generate probability estimates.^6^ This study provides additional evidence for the use of two common heuristics – representative heuristic and anchoring with adjustment heuristic – by residents when generating disease probability estimates.

So why would residents who had received training in Bayesian reasoning replace this cognitive task with heuristics?^6^ Unfortunately, answers to this question are speculative as heuristics are frequently applied subconsciously. Yet there are clearly cognitive advantages to using heuristics, such as reduced cognitive effort and the ability to still generate some estimate of probability when prevalence, likelihood ratios, and the formulae for combining these data are inaccessible.^4^,^13^ Given the limited processing capacity of working memory (where we consciously process information), using heuristics may sometime lead to better decisions than formal analysis, particularly for complex tasks.^4^,^31^,^32^ Or perhaps residents avoid Bayesian reasoning because the increased precision of probability estimates does not actually help them make clinical decisions. Proponents of *fuzzy trace theory* believe that when making decisions we create two mental representations of the data: a *verbatim* trace, which contains precise information, and a *gist* trace that is an imprecise (or *fuzzy*) representation of the data.^33^,^34^ Gist traces contain the bottom-line meaning of the data and tend to be dichotomous or ordinal (e.g., low vs. intermediate vs. high risk). Previous work has shown that even when physicians have precise clinical information, for example on the risk of cardiac disease for an individual patient, they tend to make decisions on diagnosis and treatment based upon their gist trace rather than precise information contained within the verbatim trace.^34^,^35^

There are several limitations of our study that we should highlight. This was a single-centre study with a single group of learners, both of which limit the generalizability of our findings. We studied only four target conditions and our findings may have been different had we selected more or different target conditions. Similarly, we focused on only two of a large number of possible heuristics (*representative* heuristic and *anchoring with adjustment*)^38^ and it is possible that selecting different heuristics may have altered our results. Finally, in our study design we may have introduced a performance bias by restricting the time available for diagnosing and/or denying our participants access to information technology that could facilitate Bayesian reasoning, thus increasing the likelihood of attribute substitution.

Further studies are needed to address the limitations of our study and to answer other important questions on the use of heuristics – including the impact of heuristics on diagnostic performance. Despite strongly held opinions on the merits and perils of heuristic use,^14^,^15^ previous work has suggested that both novices and experts frequently use heuristics^36^ and the findings from the literature on the impact of heuristics on diagnostic performance are mixed, with some studies suggesting that under certain circumstances using heuristics may lead to better decisions than formal analysis.^4^,^37^ Without a better understanding of how and when cognitive strategies such as heuristics impact diagnostic performance, we cannot make evidence-based recommendations on the use of different cognitive strategies.^37^

### Conclusions

In this study we found that Internal Medicine residents with prior training on Bayesian reasoning used heuristics, such as representative heuristic and anchoring with adjustment, to estimate disease probabilities. The reasons for attribute substitution in this case are unclear, and may be due to the relative cognitive ease of heuristics vs. Bayesian reasoning, or perhaps because residents in their clinical practice use gist traces rather than precise probability estimates when diagnosing. Further studies are needed to explain the reasons for attribute substitution when estimating disease probabilities and to explore the impact of additional training and resources on diagnostic decisions, such as additional training in Bayesian reasoning combined with point-of-care clinical epidemiology resources, or the use of less precise cognitive strategies.^39^–^41^
